# Growth disorders in children and adolescents affected by syndromes or diseases associated with neurodysfunction

**DOI:** 10.1038/s41598-019-52918-8

**Published:** 2019-11-11

**Authors:** Lidia Perenc, Agnieszka Guzik, Justyna Podgórska-Bednarz, Mariusz Drużbicki

**Affiliations:** 1 Institute of Physiotherapy, Medical Faculty, University of Rzeszow, Hoffmanowej 25, 35-310 Rzeszów, Poland; 2 Centre for Innovative Research in Medical and Natural Sciences, University of Rzeszow, Warzywna 1a, 35-310 Rzeszow, Poland

**Keywords:** Paediatric neurological disorders, Paediatric research, Neurodevelopmental disorders

## Abstract

We have observed that one in three patients admitted to the Neurological Rehabilitation Ward for Children and Adolescents due to a syndrome or disease associated with neurodysfunction is short of stature for their age. In order to identify the relationship between growth defects (short stature) and syndromes or diseases associated with neurodysfunction, we analyzed data collected during admission to the Neurological Rehabilitation Ward for Children and Adolescents. The study applied a retrospective analysis of data collected during hospitalization of 327 children and adolescents, aged 4–18 years, affected by congenital disorders of the nervous system and/or neurological syndromes associated with a minimum of one neurodysfunction. Two assessment systems were taken into account – one system traditionally applied, and another one in accordance with indications approved by the Food and Drug Administration, related to diagnosing short stature. The findings show more frequent co-occurrence of, as well as statistically significant correlations between, short stature in certain groups: operated myelomeningocele with hydrocephalus in the subgroup of neural tube defects (p = 0.029), tetraplegia in the subgroup of spastic cerebral palsy (p < 0.001), and hypothyroidism (p = 0.04) in the entire study group.

## Introduction

Growth is a natural biological process which depends on a number of factors such as diet, genetic determinants, hormone balance, and the presence or absence of chronic disease^1,2^. The overall health status of a child or adolescent can be monitored by measurements of body height^3^. Hence, an evaluation of a child’s body height is important for preventing developmental abnormalities as short stature in an apparently healthy child may be indicative of an undiagnosed illness. Diagnosis of abnormal height may permit early identification of the cause, and therapies to normalize body height can be introduced at a later time^4^.

Short stature is diagnosed if a child’s body height is 2 SD (standard deviations) below the mean in children of the same sex, age, and racial-ethnic group. Specific centile grids that take into account these parameters provide a reference model for the effects of growth process in a given individual. In the case of short stature, the related measure will fall below the 3rd centile^2^.

It is only in some cases that etiological cause of short stature can be determined^5^. Research indicates that one in five children with a body height 2 SD below the mean, and approximately 50% of children with body height 3 SD below the mean, may have a pathological etiology^6,7^.

Nervous system disorders can be with or without encephalopathy, and their symptoms manifest themselves in various neurodysfunctions. Encephalopathy is a general term for brain damage caused by factors of various origin that can lead to cognitive, behavioral and motor impairments making it difficult or impossible to start schooling^8–10^.

Much of the literature focusing on short stature discusses the prevalence of the condition in specific disease entities, related therapies, and case studies. However, to the best of our knowledge, there are no reports investigating the relationship between short stature and nervous system disorders. This study was designed to investigate the association between growth defects (short stature) and syndromes or disease entities linked with neurodysfunction, taking into account data collected during admission to the Neurological Rehabilitation Ward for Children and Adolescents.

## Material and Methods

### Participants

A retrospective analysis evaluated data collected between 2012 and 2016 from 327 children and adolescents admitted to the Neurological Rehabilitation Ward for Children and Adolescents in St. Queen Jadwiga Regional Hospital No. 2 in Rzeszow, Poland, in the Clinical Regional Rehabilitation and Education Centre. The inclusion criteria was as follows: informed consent given by both the children and their parents or legal guardians, an age of 4–18 years, presence of a congenital disorder of the nervous system or a neurological syndrome associated with a minimum of one neurodysfunction manifesting from infancy, measurements of body height, only one selected admission procedure of the participating patient, hospitalization during the years 2012–2016, complete data (diagnosis, anthropometric measurement – body height). Exclusion criteria was as follows: lack of informed consent given by both the children and their parents or legal guardians, age below 4 or above 18 years (lack of biological frame of reference), lack of a congenital disorder of the nervous system or a neurological syndrome associated with a minimum of one neurodysfunction manifesting from infancy, co-occurrence of more than one congenital disorder of the nervous system or the neurological syndrome (for instance: Down syndrome and cerebral palsy; neural tube defect and cerebral palsy; phenylketonuria and cerebral palsy), lack of measurement of body height, more than one selected admission procedure for the participating patient, hospitalization before 2012 or after 2016, and a lack of complete data (diagnosis, anthropometric measurement – body height).

During the years 2012–2016, 2,637 patients were hospitalized in the Neurological Rehabilitation Ward for Children and Adolescents in St. Queen Jadwiga Regional Hospital No. 2 in Rzeszow, Poland, at the Clinical Regional Rehabilitation and Education Centre (KRORE). Of these, 327 patients met inclusion criteria and were selected for the study. The retrospective analysis took into account 327 children (143 girls - 43.7%, 184 boys - 56.3%). The mean age of participants in the study group was 9.7 ± 4.3 years (median 9.0 years, the youngest being 4 years of age, the oldest at 18 years of age).

Approval to conduct the study was obtained from the Bioethics Commission at the University of Rzeszow, Poland, and the methods were performed in accordance with the relevant guidelines and regulations. Informed consent was obtained both from the children and their parents or legal guardians and, additionally, from the director of the hospital. The informed consents were collected before submitting the application to the bioethics committee.

### Procedures and data analyses

The retrospective analysis took into account basic data (age, sex, principal and additional diagnosis, body height - Ht) acquired upon patient admission. The principal and additional diagnoses were determined by various specialists (neurologists, geneticists, endocrinologists, and others) prior to hospitalization at KRORE, and anthropometric measurements were performed in the KRORE admission room, following the principles adopted in the hospital. Patients were affected by various diseases or syndromes associated with nervous system damage. These were all congenital disorders and/or conditions associated with motor defects (neurodysfunctions) persisting from infancy. The conditions presented with or without encephalopathy. Based on the criteria proposed in the literature (suspected presence or suspected lack of encephalopathy, its etiopathogenesis and nature)^11^, the patients were divided into subgroups (Table 1A). Two assessment criteria systems were taken into account to identify the patients with short stature, normal body height, or tall stature. One system is traditionally applied in clinical practice and the proposed system is based on indications related to diagnosing short stature approved by the Food and Drug Administration (FDA)^12^. To perform an assessment based on all the above criteria, a z-score for body height was calculated for each person (z-score Ht). Normative values published earlier^13^ were applied as a reference. This approach has been used previously^14^. The analyses examined the relationships between the co-occurring growth defects, in particular short stature, and the neurodysfunction diagnostic subgroups. The analyses also took into account additional diagnoses (symptomatic epilepsy as opposed to the genetically conditioned epilepsy syndrome, and hypothyroidism). Adjusted Standardized Residuals (ASR) were calculated. Values > 1.96 reflect a greater number, and those below <−1.96 correspond to a smaller number than a random distribution. In order to assess to what extent the differences between the groups reflect regularities in the target population, or whether they are random, methods of statistical inference were applied. The nominal nature of the characteristics subject to comparisons determined the choice of chi-square test of independence. Nominal regression was applied to examine the relationships between the variables: dependent qualitative and independent quantitative. Statistical significance was assumed at p < 0.05. Pearson’s Contingency Coefficient C (Cp) can only take positive values (Cp ≥ 0). When Cp is distant from 0, it reflects some relationship, and values approaching 1 correspond to a perfect association.

**Table 1:** Characteristics of the study group and growth disorders.

Diseases and syndromes associated with neurodysfunction (Principal diagnosis)			Classification with regard to etiopathogenesis, presence and character encephalopathy			Classification with regard to presence and character of encephalopathy		
	N	%		N	%		N	%
Characteristics of 327 patients with neurodysfunction								
NBIA-MPAN, Neurodegeneration with Brain Iron Accumulation –Mitochondrial Protein Associated Neurodegeneration	2	0.6	MD, metabolic disorder	7	2.1	PE, progressive encephalopathy	8	2.4
GSD II, Pompe’s disease	1	0.3						
LCHAD, long-chain 3-hydroxyacyl-coenzyme A dehydrogenase deficiency	1	0.3						
SLO, Smith-Lemli-Opitz syndrome	1	0.3						
GLUT1d, glucose transporter 1 deficiency	1	0.3						
NKH, nonketotic hyperglycinemia	1	0.3						
SMEI, Dravet’s syndrome	1	0.3	EE, epileptic encephalopathy	1	0.3			
sasMMC&HCP, state following surgery due to lumbar myelomeningocele with hydrocephalus	17	5.2	NTDs, neural tube defects	24	7.3	NPE, non-progressive encephalopathy	287	88.1
sasMMC, state following surgery due to lumbar myelomeningocele	3	0.9						
sasMM, state following surgery due to parieto-occipital meningocele	1	0.3						
ACM, Arnold-Chiari malformation	2	0.6						
HCP, isolated hydrocephalus	1	0.3						
DS, Down’s syndrome	11	3.4	GD, genetic disorders	23	7.0			
ES, Edwards syndrome	1	0.3						
PMS, Phelan-McDermid syndrome	2	0.6						
MWS, Mowat-Wilson syndrome	1	0.3						
AS, Angelman syndrome	1	0.3						
DGS, Di George syndrome	1	0.3						
46,XY,del(X)(q24)	1	0.3						
CdLS, Cornelia de Lange syndrome	1	0.3						
SDS, Schwachman-Diamond syndrome	1	0.3						
PWS, Prader-Willi syndrome	1	0.3						
46 XX, add(2)(q25)	1	0.3						
46XX, del (12) (q24.21q24.23)	1	0.3						
FAS, fetal alcohol syndrome	1	0.3	TE, toxic encephalopathy	1	0.3			
CP, cerebral palsy	239	73.1	CP cerebral palsy	239	73.1			
HMSN, hereditary motor and sensory polyneuropathy	8	2.4	NMD, neuromuscular disorders	32	9.8	NMD, neuromuscular disorders	32	9.8
LGMD, muscular dystrophy limb-girdle	7	2.1						
BMD, Becker’s muscular dystrophy	3	0.9						
DMD, Duchenne muscular dystrophy	7	2.1						
TD, Thomsen disease	1	0.3						
AMC&N arthrogryposis multiplex congenita with neuropathy	3	0.9						
CM, congenital myopathy	1	0.3						
SMA, spinal muscular atrophy	2	0.6						
B. Numerical characteristics of z-score Ht								
z-score	N	Mean	Median	SD	25-th centile	75-th centile	Min	Max
z-score Ht	327	−1.23	−1.16	1.98	−2.33	−0.05	−8.93	4.20
C. Growth defects – criteria systems								
Height			Traditional classification (Ht)			Proposed classification (Ht)		
Short stature			z-score Ht<-2			*z-score Ht<-2.25		
Normal body height			-2≥z -score Ht≤ 2			**-2.25≥z -score Ht≤ 2.25		
Tall stature			z-score Ht>2			**z-score Ht>2.25		
*FDA criterion for diagnosing short stature, **criterion proposed by this author as a modification enabling complete analyses of the acquired material								
D. Prevalence of growth defects								
Traditional classification (Ht)			Height			Proposed classification (Ht)		
N		%				N		%
97		29.7	Short stature			90		27.5
220		67.3	Normal body height			236		72.2
10		3.0	Tall stature			1		0.3

**N** - numbers of patients, **%** - percent, **z-score Ht** - z-score of height, SD - standard deviation, Min - minimum value, Max - maximum value.

## Results

Patients were divided into seven subgroups. These included six subgroups representing medical conditions usually associated with encephalopathy: progressive metabolic disorders (MD) (2.1%), progressive genetically-determined epileptic syndromes (EE) (0.3%), non-progressive neural tube defects (NTDs) (7.3%), non-progressive genetic disorders (GD): chromosomal aberrations, monogenic disorders except neuromuscular diseases (7.0%), non-progressive toxic (TE) (0.3%), and non-progressive cerebral palsy (CP) (73.1%). There was additionally one subgroup representing conditions usually not associated with encephalopathy, i.e. neuromuscular diseases (NMD) (9.8%) (Table 1A). Taking into account the character and expected presence of encephalopathy^11^, the smaller subgroups were combined into two larger groups representing progressive encephalopathy (PE) (2.4%) and non-progressive encephalopathy (NPE) (88.1%). The third group represented neuromuscular diseases (NMD) (9.8%). The internal structure of subsequent subgroups was differentiated due to various diagnoses. In view of the variability of neural tube defects^15^ and the key importance of further operative treatment^16^, the patients in the NTDs subgroup were divided into those operated on due to myelomeningocele with hydrocephalus (sasMMC&HCP), and those operated on exclusively due to myelomeningocele (sasMMC). The presentation also included the remaining cases where no operative treatment was applied. The subgroup with GD included both chromosomal aberrations and genetic mutations. It should be mentioned that some authors point to chromosomal disorders and genetic mutations as causes of short stature^17,18^. Down syndrome and Prader-Willi syndrome also belong to this group, alongside with other genetically determined diseases, including mutations of a single gene^17,18^.

Among all patients, those with CP constituted the largest group (73.1%). Analysis of the diagnoses showed the following types of CP, as proposed by Hagber^11^: spastic – 93.7% (N = 223), mixed 5% (N = 12), ataxic 1.7% (N = 4). No cases of dyskinetic type were identified. Among those with spastic CP, 34.1% (N = 76) presented with tetraplegia, 40.4% with diplegia (N = 90), and 25.6% with hemiplegia (N = 57). Principal diagnoses were accompanied with additional diagnoses: symptomatic epilepsy 26.3% (N = 86), and hypothyroidism 4.3% (N = 14).

The mean and the median for z-score Ht in the study group assumed values lower than −1, and higher than −2. Based on two assessment criteria – one system traditionally applied in clinical practice and the other based on indications approved by the Food and Drug Administration (FDA)^2^, related to diagnosing short stature – patients were divided into those with normal body height, short stature and tall stature (Table 1B,C).

In accordance with the traditional classification, normal body height was found in 67% of the patients, short stature in 30% and tall stature in 3% of the patients. Short stature was observed in patients with: NBIA-MPAN (N = 1), sasMMC&HCP (N = 10), ACM (N = 1), DS (N = 6), ES (N = 1), DGS (N = 1), CdLS (N = 1), FAS (N = 1), CP (N = 63), HMSN (N = 3), LGMD (N = 1), DMD (N = 4), AMC&N (N = 2), CM (N = 1), SMA (N = 1) (Table 2A). Tall stature was found in sasMMC&HCP (N = 1), HCP (N = 1), CP (N = 7) and BMD (N = 1). The assessment based on FDA criteria showed short stature at a rate of 28% (Table 1D).

**Table 2:** Traditional body height classification (Ht) and A. units and syndromes associated with neurodysfunction, B. classification with regard to etiopathogenesis, presence and character of encephalopathy

A. Units and syndromes running with neurodysfunction	Normal body height		Short stature		Tall stature		Total
	N (%)	ASR	N (%)	ASR	N (%)	ASR	N (%)
**Height – traditional classification (Ht) by z-score Ht (*p*** = **0.172**; **Cp** = **0.574)**							
NBIA-MPAN	1 (50%)	−0.5	1 (50%)	0.6	0	−0.3	2
GSD II	1	0.7	0	−0.7	0	−0.2	1
LCHAD	1	0.7	0	−0.7	0	−0.2	1
SLO	1	0.7	0	−0.7	0	−0.2	1
GLUT1d	1	0.7	0	−0.7	0	−0.2	1
NKH	1	0.7	0	−0.7	0	−0.2	1
SMEI	1	0.7	0	−0.7	0	−0.2	1
sasMMC&HCP	6 (35%)	−2.9	10 (59%)	2.7	1 (6%)	0.7	17
sasMMC	3	1.2	0	−1.1	0	−0.3	3
sasMM	1	0.7	0	−0.7	0	−0.2	1
ACM	1 (50%)	−0.5	1 (50%)	0.5	0	−0.3	2
HCP	0	−1.4	0	−0.7	1	5.6	1
DS	5 (45%)	−1.6	6 (55%)	1.8	0	−0.6	11
ES	0	−1.4	1	1.5	0	−0.2	1
PMS	2	1.0	0	−0.9	0	−0.3	2
MWS	1	0.7	0	−0.7	0	−0.2	1
AS	1	0.7	0	−0.7	0	−0.2	1
DGS	0	−1.4	1	1.5	0	−0.2	1
46, XY, del(X) (q24)	1	0.7	0	−0.7	0	−0.2	1
CdLS	0	−1.4	1	1.5	0	−0.2	1
SDS	1	0.7	0	−0.7	0	−0.2	1
PWS	1	0.7	0	−0.7	0	−0.2	1
46 XX, add(2) (q25)	1	0.7	0	−0.7	0	−0.2	1
46XX, del (12) (q24.21q24.23)	1	0.7	0	−0.7	0	−0.2	1
FAS	0	−1.4	1	1.5	0	−0.2	1
CP	169 (71%)	2.2	63 (26%)	−2.2	7 (3%)	−0.2	239
HMSN	5 (63%)	−0.3	3 (37%)	0.5	0	−0.5	8
LGMD	6 (86%)	1.1	1 (14%)	−0.9	0	−0.5	7
BMD	2 (67%)	0.0	0	−1.1	1 (33%)	3.1	3
DMD	3 (43%)	−1.4	4 (57%)	1.6	0	−0.5	7
TD	1	0.7	0	−0.7	0	−0.2	1
AMC&N	1 (33%)	−1.3	2 (67%)	1.4	0	−0.3	3
CM	0	−1,4	1	1.5	0	−0.2	1
SMA	1 (50%)	−0.5	1 (50%)	0.6	0	−0.3	2
Total	220 (67%)		97 (30%)		10 (3%)		327
**B. Classification with regard to etiopathogenesis**, **presence and character of encephalopathy**	**Normal body height**		**Short stature**		**Tall stature**		**Total**
	**N (%)**	**ASR**	**N (%)**	**N (%)**	**ASR**	**N (%)**	**N (%)**
**Height – traditional classification (Ht) by z-score Ht (*p*** = **0**.**318; Cp = 0**.**201)**							
MD	6 (86%)	1.1	1 (14%)	−0.9	0	−0.5	7
EE	1	0.7	0	−0.7	0	−0.2	1
NTDs	11 (46%)	−2.3	11 (46%)	1.8	2 (8%)	1.6	24
GD	14 (61%)	−0.7	9 (39%)	1.0	0	−0.9	23
TE	0	−1, 4	1	1.5	0	−0.2	1
CP	169 (71%)	2.2	63 (26%)	−2.2	7 (3%)	−0.9	239
NMD	19 (59%)	−1.0	12 (38%)	1.0	1 (3%)	0.0	32
Total	220 (67%)		97 (30%)		10 (3%)		327

N – numbers of patients, % - percent, *p* –probability value calculated by chi-square test of independence, Cp – Pearson’s Contingency Coefficient C, Cp ≥ 0, values distant from 0 reflect some relationship; values approaching 1 correspond to a perfect association., ASR - Adjusted Standardized Residuals, values >1.96 reflect a greater number, and those below <−1.96 correspond to a smaller number than a random distribution.

In the next stage, the analyses examined the relationships between the co-occurring growth defects, in particular short stature, and the diseases or syndromes associated with neurodysfunction, relative to the identified subgroups and within the identified subgroups.

The findings show no more/less frequent (%) co-occurrence and no statistically significant correlations between:

- Proposed body height classification (Ht) and classification with regard to etiopathogenesis, presence and character encephalopathy (Table 3B).

**Table 3:** Proposed body height classification (Ht) and A. units and syndromes associated with neurodysfunction, B. classification with regard to etiopathogenesis, presence and character of encephalopathy.

A. Units and syndromes associated with neurodysfunction	Normal body height		Short stature		Tall stature		Total
	N (%)	ASR	N (%)	ASR	N (%)	ASR	N (%)
**Height - proposed classification (Ht) by z-score Ht (*p* = 0**.**997; Cp** = **0**.**324)**							
NBIA-MPAN	1 (50%)	−0.7	1 (50%)	0.5	0	−0.1	2
GSD II	1	0.6	0	−0.6	0	−0.1	1
LCHAD	1	0.6	0	−0.6	0	−0.1	1
SLO	1	0.6	0	−0.6	0	−0.1	1
GLUT1d	1	0.6	0	−0.6	0	−0.1	1
NKH	1	0.6	0	−0.6	0	−0.1	1
SMEI	1	0.6	0	−0.6	0	−0.1	1
sasMMC&HCP	8 (47%)	−2.9	9 (53%)	2.4	0	−0.2	17
sasMMC	3	1.1	0	−1.1	0	−0.2	3
sasMM	1	0.6	0	−0.6	0	−0.1	1
ACM	1 (50%)	−0.7	1 (50%)	0.7	0	−0.1	2
HCP	1	0.6	0	−0.6	0	−0.1	1
DS.	6 (55%)	−1.3	5 (45%)	1.4	0	−0.2	11
ES	0	−1.6	1	1.6	0	−0.1	1
PMS	2	0.9	0	−0.9	0	−0.1	2
MWS	1	0.6	0	−0.6	0	−0.1	1
AS	1	0.6	0	−0.6	0	−0.1	1
DGS	0	−1.6	1	1.6	0	−0.1	1
46, XY, del(X) (q24)	1	0.6	0	−0.6	0	−0.1	1
CdLS	0	−1.6	1	1.6	0	−0.1	1
SDS	1	0.6	0	−0.6	0	−0.1	1
PWS	1	0.6	0	−0.6	0	−0.1	1
46 XX, add(2) (q25)	1	0.6	0	−0.6	0	−0.1	1
46XX, del (12) (q24.21q24.23)	1	0.6	0	−0.6	0	−0.1	1
FAS	0	−1.6	1	1.6	0	−0.1	1
CP	179 (75%)	1.8	59 (24.5%)	−1.9	1 (0.5%)	0.6	239
HMSN	6 (75%)	0.2	2 (25%)	−0.2	0	−0.2	8
LGMD	6 (86%)	0.8	1 (14%)	−0.8	0	−0.1	7
BMD	3	1.1	0	−1.1	0	−0.1	3
DMD	3 (43%)	−1.7	4 (57%)	1.8	0	−0,1	7
TD	1	0.6	0	−0.6	0	−0.1	1
AMC&N	1 (33%)	−1.5	2 (67%)	1.5	0	−0.1	3
CM	0	−1.6	1	1.6	0	−0.1	1
SMA	1 (50%)	−0.7	1 (50%)	0.7	0	−0.1	2
Total	236 (72%)		90 (27.5%)		1 (0.5%)		327
**B**. **Classification with regard to etiopathogenesis**, **presence and character of encephalopathy**	**Normal body height**		**Short stature**		**Tall stature**		**Total**
	**N (%)**	**ASR**	**N (%)**	**ASR**	**N (%)**	**ASR**	**N (%)**
**Height - proposed classification (Ht) by z-score Ht (*p*** = **0**.**732; Cp = 0**.**161)**							
MD	6 (86%)	0.8	1 (14%)	−0.8	0	−0.1	7
EE	1	0.6	0	−0.6	0	−0.1	1
NTDs	14 (58%)	−1.6	10 (42%)	1.6	0	−0.3	24
GD	15 (65%)	−0.8	8 (35%)	0.8	0	−0.3	23
TE	0	−1.6	1	1.6	0	−0.1	1
CP	179 (75%)	1.8	59 (24.5%)	−1.9	1 (0.5%)	0.6	239
NMD	21 (66%)	−0.9	11 (34%)	0.9	0	−0.3	32
Total	236 (72%)		90 (27.5%)		1 (0.5%)		327

N – numbers of patients, % - percent, *p* –probability value calculated by chi-square test of independence, Cp – Pearson’s Contingency Coefficient C, Cp ≥ 0, values distant from 0 reflect some relationship; values approaching 1 correspond to a perfect association, ASR - Adjusted Standardized Residuals, values >1.96 reflect a greater number, and those below <−1.96 correspond to a smaller number than a random distribution.
- Proposed body height classification (Ht) and NTDs (Table 4B).

**Table 4:** Growth disorders and: NDTs (A-B), type of spasticity in CP (C–F).

A. NTDs	Normal body height		Short stature		Tall stature		Total
	N (%)	ASR	N (%)	ASR	N (%)	ASR	N (%)
**Height - traditional classification (Ht) by z-score Ht (*p*** = **0**.**029; Cp** = **0**.**645)**							
sasMMC&HCP	6 (35%)	−1.6	10 (59%)	2.0	1 (6%)	−0.7	17
sasMMC	3	2.0	0	−1.7	0	−0.6	3
sasMM	1	1.1	0	−0.9	0	−0.3	1
ACM	1 (50%)	0.1	1 (50%)	0.1	0	−0.4	2
HCP	0	−0.9	0	−0.9	1	3.4	1
In Total	11 (46%)		11 (46%)		2 (8%)		24
**B**. **NTDs**	**Normal body height**		**Short stature**		**Tall stature**		**Total**
	**N (%)**	**ASR**	**N (%)**	**ASR**	**N (%)**	**ASR**	**N (%)**
**Height - proposed classification (Ht) by z-score Ht (*p*** = **0**.**340; Cp** = **0**.**398)**							
sasMMC&HCP	8 (47%)	−1.7	9 (53%)	1.7	0	—	17
sasMMC	3	1.6	0	−1.6	0	—	3
sasMM	1	0.9	0	−0.9	0	—	1
ACM	1 (50%)	−0.2	1 (50%)	0.2	0	—	2
HCP	1	0.9	0	−0.9	0	—	1
Total	10 (42%)		14 (58%)		0		24
**C**. **Type of spasticity (CP)**	**Normal body height**		**Short stature**		**Tall stature**		**Total**
	**N (%)**	**ASR**	**N (%)**	**ASR**	**N (%)**	**ASR**	**N (%)**
**Height - traditional classification (Ht) by z-score Ht (*p*** < **0**.**001; Cp=0**.**328)20**							
Diplegic	67 (75%)	1,2	21 (23%)	−1,0	2 (2%)	−0,6	90
Hemiplegic	50 (88%)	3,4	4 (7%)	−3,9	3 (5%)	1,1	57
Tertaplegic	39 (51%)	−4,4	35 (46%)	4,6	2 (3%)	−0,3	76
Total	156 (70%)		60 (27%)		7 (3%)		223
**D**. **Type of spasticity (CP)**	**Normal body height**		**Short stature**		**Tall stature**		**Total**
	**N (%)**	**ASR**	**N (%)**	**ASR**	**N (%)**	**ASR**	**N (%)**
**Height - proposed classification (Ht) by z-score Ht (*p*** < **0**.**001; Cp** = **0**.**314)21**							
Diplegic	70 (78%)	0.9	20 (22%)	−0.8	0	−0.8	90
Hemiplegic	53 (93%)	3.7	4 (7%)	−3.7	0	−0.6	57
Tetraplegic	43 (57%)	−4.4	32 (42%)	4.2	1 (1%)	1.4	76
Total	166 (74.5%)		56 (25%)		1 (0.5%)		223
**E**. **Type of spasticity (CP)**	**Normal body height**		**Short stature**		**Tall stature**		**Total**
	**N (%)**	**ASR**	**N (%)**	**ASR**	**N (%)**	**ASR**	**N (%)**
**Height - traditional classification (h) by z-score Ht (*p*** < **0**.**001; Cp** = **0**.**297)**							
Tetraplegic	39 (51%)	−4,4	35 (46%)	4.6	2 (3%)	−0.3	76
Others: diplegic, hemiplegic	117 (77%)	4,4	25 (17%)	−4.6	5 (3%)	0.3	147
Total	156 (70%)		60 (27%)		7 (3%)		223
**F**. **Type of spasticity (CP)**	**Normal body height**		**Short stature**		**Tall stature**		**Total**
	**N (%)**	**ASR**	**N (%)**	**ASR**	**N (%)**	**ASR**	**N (%)**
**Height - proposed classification (h) by z-score Ht (*p*** < **0**.**001; Cp** = **0**.**288)**							
Tetraplegia	43 (57%)	−4.4	32 (42%)	4.2	1 (1%)	1.4	76
Others: diplegic, hemiplegic	123 (84%)	4.4	24 (16%)	−4.2	0	−1.4	147
Total	166 (74.5%)		56 (25%)		1 (0.5%)		223

N – numbers of patients, % - percent, *p* –probability value calculated by chi-square test of independence, Cp – Pearson’s Contingency Coefficient C,Cp ≥ 0, values distant from 0 reflect some relationship; values approaching 1 correspond to a perfect association, ASR - Adjusted Standardized Residuals, values > 1.96 reflect a greater number, and those below <−1.96 correspond to a smaller number than a random distribution.
- Hypothyroidism and tetraplegia (Table 5E).

**Table 5:** Growth disorders, z-score Ht and hypothyroidism (A,B,D), z-score Ht and tetraplegia (C), hypothyroidism and tetraplegia (E).

A. Accompanying recognition Hypothyroidism	Normal body height		Short stature		Tall stature		Total
	N (%)	ASR	N (%)	ASR	N (%)	ASR	N (%)
**Height - traditional classification (h) by z-score Ht (*p*** = **0.065; Cp = 0.128)**							
Present	6 (43%)	−2.0	8 (57%)	2.3	0	−0.7	14
Lack	214 (68.5%)	2.0	89 (28.5%)	−2.3	10 (3%)	0.7	313
Total	220 (67%)		97 (30%)		10 (3%)		327
**B. Accompanying recognition Hypothyroidism**	**Normal body height**		**Short stature**		**Tall stature**		**Total**
	**N (%)**	**ASR**	**N (%)**	**ASR**	**N (%)**	**ASR**	**N (%)**
**Height - proposed classification (h) by z-score Ht (*p*** = **0**.**040; Cp** = **0**.**139)**							
Present	6 (43%)	−2.5	8 (57%)	2.5	0	−0.2	14
Lack	230 (73.5%)	2.5	82 (26%)	−2.5	1 (0.5%)	0.2	313
Total	236 (72%)		90 (27.5%)		1 (0.5%)		327
**C**. **Relationship between CP spastic type and z-score Ht**							
Nominal regression			Quantitative dependent variable z-score Ht				
*Qualitative dependent variable* Type of spasticity (CP)	Tetraplegia (34.1%)		<0.001				*p*
			0.689 0.581–0.816				*OR*
	Other: diplegia, hemiplegia (65.9%)						*Reference group*
**D**. **Relationship between hypothyroidism and z-score Ht**							
Nominal regression			Quantitative dependent variable z-score Ht				
*Qualitative dependent variable* Accompanying recognition	Presence of hypothyroidism (4.5%)		0.014				*p*
			0.739 0.581–0.941				*OR*
	Lack of hypothyroidism (95.7%)						*Reference group*
**E**. **Accompanying recognition** **Hypothyroidism**	**Tetraplegia**		**Diplegia**		**Hemiplegia**		**Total**
	**N (%)**	**ASR**	**N (%)**	**ASR**	**N (%)**	**ASR**	**N (%)**
**Type of spasticity (CP) (*p*** = **0**.**450; Cp** = **0**.**084)**							
Present	3 (60%)	−1.2	1 (20%)	−0.9	1 (20%)	−0.3	5
Lack	73 (33%)	1.2	89 (41%)	0.9	56 (26%)	0.3	218
Total	76 (34%)		90 (40%)		57 (26%)		223

**N** – numbers of patients, **%** - percent, ***p*** –probability value calculated by chi-square test of independence, **Cp** – Pearson’s Contingency Coefficient C,Cp ≥ 0, values distant from 0 reflect some relationship; values approaching 1 correspond to a perfect association, **ASR** - Adjusted Standardized Residuals, values > 1.96 reflect a greater number, and those below <−1.96 correspond to a smaller number than a random distribution, **OR –** Odds Ratio (95% confidence interval).
- The findings show more/less frequent (%) co-occurrence and no statistically significant correlations between:
- Traditional body height classification (Ht) and units and syndromes associated with neurodysfunction. Normal body height more frequently co-occurs with CP (71%), less frequently with sasMMC&HCP (36%). Short stature more frequently co-occurs with sasMMC&HCP (N% = 59%), less frequently with CP (26%), and tall stature more frequently co-occurs with BMD (33%) and HCP (100%). However, the correlation is not statistically significant (p = 0.172) (Table 2A).
- Proposed body height classification (Ht) and units and syndromes associated with neurodysfunction. Normal body height less frequently co-occurs with sasMMC&HCP (47%), and short stature more frequently co-occurs with sasMMC&HCP (53%). The correlation is not statistically significant (p = 0.997) (Table 3A).
- Traditional body height classification (Ht) and classification with regard to etiopathogenesis, presence and character encephalopathy. Normal body height more frequently co-occurs with CP (71%), and less frequently with NTDs (46%). Short stature less frequently co-occurs with CP (26%). The correlation is not statistically significant (p = 0.318) (Table 2B).
- Traditional body height classification (Ht) and hypothyroidism. Normal body height co-occurs more frequently with lack of hypothyroidism (68%), and less frequently with existing hypothyroidism (43%). Short stature more frequently co-occurs with hypothyroidism (57%), and less frequently with lack of hypothyroidism (28%). The correlation is not statistically significant (p = 0.065) (Table 5A).
- In accordance with the traditional classification, normal body height was found in 67% of patients, short stature in 30%, and tall stature in 3% of patients. Assessment based on FDA criteria showed short stature at the rate of 28% (Table 2C).
- The findings show more/less frequent (%) co-occurrence as well as statistically significant relations between:
- Traditional body height classification (Ht) and NTDs. Short stature more frequently co-occurs with sasMMC&HCP (59%). Normal body height is more common in sasMMC (100%). Tall stature occurs more frequently in isolated HCP (100%). The relation is statistically significant (p = 0.029) (Table 4A).
- Traditional body height classification (Ht) and CP spastic type. Short stature more frequently co-occurs with tetraplegia (46%), and less frequently with hemiplegia (7%). Normal body height is more common in hemiplegia (88%), and less common in tetraplegia (51%). The relation is statistically significant (p < 0.001) (Table 4B).
- Proposed body height classification (Ht) and type of spasticity. Short stature again more frequently co-occurs with tetraplegia (42%), and less frequently with hemiplegia (7%). Normal body height is more common in hemiplegia (93%), and less common in tetraplegia (57%). The relation is statistically significant (p < 0.001) (Table 4C). The traditional classification is slightly more effective in differentiating this relationship (Cp = 0.328) compared to the proposed classification (Cp = 0.314) – Table 4B,C.
- Traditional body height classification (Ht) and type of spasticity: tetraplegic and other (i.e. diplegic, hemiplegic). The relation is statistically significant (p < 0.001) (Table 4D).
- Proposed body height classification (Ht) and type of spasticity: tetraplegic and other (i.e. diplegic, hemiplegic). In the group of children and adolescents with spastic CP, short stature frequently co-occurs with tetraplegia (Table 4E). Again, the traditional classification is slightly more effective in differentiating this relationship (Cp = 0.297) compared to the proposed classification (Cp = 0.288) – Table 4D,E.
- Proposed body height classification (Ht) and hypothyroidism. Normal body height frequently co-occurs with a lack of hypothyroidism (74%), and is less common in the patients with hypothyroidism (43%). Short stature frequently co-occurs with hypothyroidism (57%), and is less common in the patients without hypothyroidism (26%). The relationship is statistically significant (p = 0.040). By using the FDA criterion for diagnosing short stature it was possible to show frequent co-occurrence of short stature and hypothyroidism in the group of children and adolescents with congenital disorders of the nervous system and/or neurological syndromes with symptoms manifesting from infancy (Table 5B).

In the remaining cases the findings showed no statistically significant relations. In the next stage the analyses examined relations between the quantitative independent variable, i.e. z-score Ht, and a dependent qualitative variable: distinctions within subgroups or presence of an additional diagnosis. Statistical significance was identified in two conditions, i.e. division of the spastic CP type subgroup into tetraplegia and other (i.e. diplegia, hemiplegia); as well as division of the whole group of patients relative to presence or lack of hypothyroidism. Tetraplegia co-occurs with lower values of z-score Ht (p < 0.001, OR = 0.689). Tetraplegia is more likely to have low z-scores Ht than other spastic CP types (Table 5C). Hypothyroidism also co-occurs with lower values of z-score Ht (p = 0.014, OR = 0.739). Hypothyroidism is more likely to have low z-score Ht than cases without hypothyroidism (Table 5D).

## Discussion

Low stature affects about 2% of the general population of children and adolescents and represents one of the more common developmental abnormalities and the reasons for consulting a pediatric endocrinologist^17^. In the studied group of children and adolescents hospitalized in the rehabilitation department due to neurodysfunction, a higher incidence of short stature was found to be 29.7% (traditional criterion) or 27.5% (criterion accepted by the FDA). Normal body height is an important measure of health and well-being in children while growth disorders may result from destabilized diet, environment or health of the child^19,20^. Dysfunctions in a child’s nervous system may be caused by numerous factors: genetic, contagious physical, chemical or environmental. Prevalence of neurodysfunctions in pediatric populations is relatively high, compared to defects of other systems. This is linked with the fact that development of the specific structures of the nervous system occurs over a relatively long period of time, during which the processes are affected by harmful factors. It may be difficult to accurately identify the cause even in half of the children affected by the condition^21–23^. Research has shown that dysfunctions of the nervous system may impact body height. In fact literature review shows that there are some publications focusing on growth disorders in children with CP^19,24–27^, with NTDs^28^, with SD^29^ or with hypothyroidism^8,30^, however there are no studies reporting cumulative analyses of relationships between co-occurring growth disorders and syndromes as well as diseases associated with neurodysfunction. Hence, this article presents the first scientific report related to this area.

Coexistence of short stature and major diagnoses was examined, however no statistical significance was obtained. Short stature was observed in patients with: NBIA-MPAN, sasMMC&HCP, ACM, DS, ES, DGS, CdLS, FAS, CP, HMSN, LGMD, DMD, AMC&N, CM, and SMA. Therefore, subgroups were distinguished among the patients studied. The coexistence of short stature and separated subgroups as well as short stature and diagnoses in subgroups was studied.

The current study shows more frequent co-occurrence of, and statistically significant relationships between short stature (based on the traditional classification as well as on FDA criteria) and tetraplegia in subgroup with spastic CP. It was also shown that tetraplegia co-occurs with the lower z-score Ht. Growth disorder is a common secondary impairment in children with CP^19^. Likewise, children with CP grow more slowly than children with no chronic conditions, despite appropriate environment and regularly provided medical care^31^. Growth disparities in CP increase when children become older^24^. Affected limbs in children with spastic hemiparesis due to CP are characterized by delayed skeletal maturation and decreased bone density, compared to unaffected limbs, and this may be linked with growth defects in this group of patients^25^. Stallings *et al*. point out that growth disparities may be observed in all children with CP^26^, however research shows that frequency of growth impairments increases with greater motor disabilities^24,25,27^. Melunovic *et al*. examined anthropometric parameters, including body height, in 80 children with CP, and compared these with normal values in healthy children, and relative to the degree of motor disability. They showed significant differences in all the monitored parameters with respect to normative values, where 63% of the children with CP presented severe motor impairment^25^. These findings have been confirmed in the present study which shows statistically significant relationship between growth and tetraplegia.

An important finding of the current study is the higher prevalence of short stature (traditional classification) in the children with sasMMC&HCP among patients with NTDs. The problem of short stature in children with neural tube defects has been widely known for a long time. Short stature in this group of patients is attributed to smaller lower limbs, spinal deformities and scoliosis^28^.

As it is known, hypothyroidism is one of many causes of short stature^17^. Besides the main diagnoses, we took into consideration additional diagnoses such as hypothyroidism. In Poland, thyroid disease problems are very rarely investigated^32^. In fact, hypothyroidism is the most common thyroid disease. It is estimated that about 2–5% of people in Poland suffer from it. The incidence of hypothyroidism increases with age^32,33^. In the group of children and adolescents examined, hypothyroidism was as common as in the general population - with a rate of 4.3%. It has been shown that, similarly to the general population, in children and adolescents with neurodysfunction, short stature and hypothyroidism occur together. It was also demonstrated that hypothyroidism co-occurs with lower values of z-score Ht. Hypothyroidism may mask other concomitant causes of short stature. In the absence of a satisfactory growth effect during levothyroxine therapy, other possible causes of short height should be investigated^17^. It is noteworthy that all the patients were treated for hypothyroidism. It should be mentioned that hypothyroidism by itself cannot explain such frequent incidence of short stature in the relevant group of children.

Hypothyroidism in children and adolescents is a frequent problem in developing parts of the world and the condition leads to short stature^30^. Gutch *et al*. in their study assessed the prevalence of short stature in adolescents with hypothyroidism. Out of 900 patients with hypothyroidism, 87 subjects aged 6–18 years were enrolled for the study; they were newly diagnosed with hyperthyroidism or had been treated in an endocrine clinic for up to 1.5 years. The mean age for hypothyroidism diagnosis in the adolescents was 11.2 years. Short stature was observed in 45%, and delayed bone age was found in 72% of the subjects. The authors point out that prompt diagnosis of hypothyroidism may lead to early and effective treatment, and consequently to improvements related to skeletal defects and short stature^30^. Hussein *et al*. assessed frequency of etiological factors leading to short stature in a group of 637 children (354 boys and 283 girls). Hypothyroidism was the second most common endocrine factor for short stature, preceded only by growth hormone deficiency^8^.

The present study shows that among children and adolescents with neurodysfunction there are relationships between short stature and NTDs, as well as between short stature and spastic CP. Furthermore, short stature in the study group co-occurs with hypothyroidism. It is necessary to carry out endocrinological diagnostics in children and adolescents who have neurodysfunction. Short stature cannot be explained by just “innervation disorders” linked to abnormalities in the central and peripheral nervous system.

## Conclusions

Children and adolescents with neurodysfunction present growth defects.

Short stature in children and adolescents with neurodysfunction co-occurs with hypothyroidism over the whole group studied, tetraplegia in the subgroup with spastic CP, and in patients operated on due to myelomeningocele with hydrocephalus belonging to the NTDs.
